# Blocking Tics in Gilles de la Tourette Syndrome

**DOI:** 10.3389/fneur.2021.686785

**Published:** 2021-05-31

**Authors:** Justyna Kaczyńska, Piotr Janik

**Affiliations:** Department of Neurology, Medical University of Warsaw, Warsaw, Poland

**Keywords:** Gilles de la Tourette syndrome, Tourette disorder, blocking tics, blocking phenomena, negative motor phenomena, complex tics, obsessive-compulsive disorder

## Abstract

**Introduction:** Patients with Gilles de la Tourette syndrome (GTS) may experience blocking tics (BTs) defined as recurrent, brief cessations of motor acts. The aim of this study was to assess the prevalence, age of onset, and clinical correlates of BTs in GTS patients.

**Materials and Methods:** We performed a one-time registration study in a cohort of 195 consecutive GTS patients aged 5–66 years (mean age: 15.0 ± 9.2; 47 females, 24.1%). All patients were personally interviewed and examined.

**Results:** At least one BT occurred at some point in the lifetime of 73 patients (37.4%) with a mean age of onset of 10.4 ± 5.9 years. BTs occurred an average of 4.8 ± 5.3 years after tic onset. The most common BT was cessation of walking (*n* = 59, 80.8%), followed by speech (*n* = 19, 26.0%), running (*n* = 18, 24.7%), and writing (*n* = 9, 12.3%). Most of the patients (*n* = 52, 71.2%) reported cessation of only one activity. Clinical associations of BTs included more severe tics, overall greater number of tics, and, to a lesser extent, higher age at evaluation and comorbid obsessive-compulsive disorder.

**Conclusions:** BTs represent complex tics, early and common symptoms of GTS, and are associated with a more severe form of GTS.

## Introduction

Tics are defined as sudden, rapid, recurrent, non-rhythmic motor movements or vocalizations, generally preceded by urges. This definition is accurate for motor clonic tics, but some tics do not fulfill the above criteria. Blocking tics (BTs) are one of those that differ from typical tics and could be classified in the spectrum of negative motor phenomena which also include negative myoclonus, freezing of gait, epilepsy and cataplexy. Blocking phenomena occur in Gilles de la Tourette syndrome (GTS) but they have not been widely investigated and only a few reports on them are available in the medical literature.

Ganos et al. (1) distinguished three types of these blocking phenomena: related to tics, related to obsessive-compulsive behavior/disorder (OCB/OCD), and of a functional nature. The authors emphasized that there are two types of blocking phenomena regarding tics: a brief inhibition of voluntary movement resulting from prolonged dystonic tics, or a cessation of motor output appearing without any noticeable reason. In the authors' experience, blocking phenomena in the context of tics are rare and usually associated with a more severe form of GTS. Jankovic (2) emphasized that BTs may result not only from prolonged dystonic but also tonic tics. Fasano et al. (3) reported about a patient who presented with complex motor tics which affected his gait. In this case a sequence of jerks involving the trunk, arms, and face was leading to sudden cessation of walking. Rizzo et al. (4) published a case report of a patient who suffered from a gait disorder in the form of episodes of sudden gait arrest. The authors eventually qualified gait disorders as tics. Many arguments supported this diagnosis: the patient experienced a typical premonitory urge when she tried to suppress gait arrest and the relief of this feeling after she stopped again, when gait arrest could manifest repeatedly in clusters. The patient also fulfilled the diagnostic criteria of GTS and therapy with risperidone was effective.

Blocking phenomena may also apply to speech and result in difficulties with speech initiation, interruption of speech, and problems with completing sentences, resembling stuttering or cluttering. Jakubovski and Müller-Vahl (5) published a description of two young male patients with treatment-resistant GTS who suffered from significant speech disfluencies caused by vocal blocking tics and palilalia. Van Borsel and Vanryckeghem (6) presented the case of an 18-year-old patient in which, at the age of 14, ~2 years after the diagnosis of GTS, disfluent speech was first observed. One of the manifestations of his speech disorder was an inability to formulate ideas, due to halting speech and then continuing speaking in short spurts. In another paper, Van Borsel et al. (7) analyzed the speech patterns of three patients with GTS. Among many disfluencies, incomplete phrases, within-word blocks, and broken words were found.

Kwak and Jankovic (8) mentioned several mechanisms underlying phonic BTs. One of them is an isometric contraction of the abdominal muscles producing a Valsalva maneuver which leads to sudden cessation of speech. Blocking tics may also be part of a sequential complex tic, and the influence of comorbid OCD is also important in the appearance of BTs. Ganos et al. (1) highlighted that the blocking phenomena associated with OCB/OCD phenomenologically resembles the cessation of motor output caused by tics, however, obsessions and covert compulsions are the reason for the cessation of the patient's activities.

According to the above literature data and our own clinical experience, we divided BTs into primary and secondary. Primary BTs are a sudden cessation of voluntary motor acts neither due to other motor tics nor vocalizations, usually not preceded by urges, while secondary BTs are a consequence of other motor tics, usually related to premonitory urges. We also noticed that some patients with GTS and comorbid OCD experienced tic-like blocks rather than OCD-related motor blocks. This suggests that in GTS patients both tics and OCD symptoms may lead to the manifestation of BTs. In contrast to Ganos et al. (1), we encountered BTs quite often in children and adults with GTS. To the best of our knowledge, there are no research data regarding BTs in clinical samples in medical literature.

The aim of this study was to assess the prevalence, age of onset, and clinical correlates of BTs. We hypothesized that: (1) the incidence of BTs is higher than previously expected and varies with the age of the patient; (2) patients with more severe tics and comorbid OCD develop BTs more often; (3) premonitory urges are experienced by patients with BTs less often as BTs occur abruptly and are more automatic than other tics.

## Materials and Methods

### Clinical Evaluation

All patients were recruited from a single outpatient clinic and were personally reviewed and evaluated by a single clinician well-experienced in tic disorders (PJ). The patients were registered in the study only once, and no additional clinical data obtained in follow-up visits were included in the analysis.

The patients were diagnosed with GTS in accordance with the *Diagnostic and Statistical Manual of Mental Disorders* (DSM-5). All patients were systematically interviewed with the aid of a semi-structured interview comprised of demographic and clinical data which was gathered for all of the patients. This questionnaire is based on the TIC (Tourette syndrome International database Consortium) Data Entry Form, developed by Freeman et al. (9), in which study the investigator (PJ) participated and subsequently uses this form in his own clinical practice. This interview had been slightly modified over time and expanded to include questions regarding different types of tics, including BTs (each symptom was scored as either present or absent).

BTs were defined as sudden and transient cessation of motor activity with maintained consciousness (2). The diagnosis of BTs was based on the interview, occurrence during the examination, or provided video recordings. We actively asked the patients if they had experienced motor blocks in the past or at present (within the last 7 days). Based on our experience, we distinguished four activities that we asked for regarding BTs phenomenon: walking, running, speech and writing. We did not analyze primary and secondary tics separately because, due to recall bias, many patients were unable to precisely describe their symptoms and did not remember whether a BT was related to another tic or not. Therefore, we combined both of these blocking phenomena into one group of BTs. We excluded OCD-related blocking phenomena resulting from severe, time-consuming obsessions or mental compulsions (e.g., counting, checking, “just right phenomenon”) which usually lasted much longer compared to BTs and were better explained by OCD symptoms. If we suspected a functional nature of blocking phenomena these patients were also excluded from the study.

The described phenomena are illustrated by the attached videos. Supplementary Video 1 shows an 11-year-old girl with multiple motor BTs. In the Supplementary Video 2 patient with severe phonic BTs is presented. The Supplementary Video 3 shows a patient with OCD-related motor blocks, and although such patients were not included into our study, the differential diagnosis between BTs and OCD-related blocking phenomena is essential.

The Yale Global Tic Severity Scale (YGTSS) was used to assess the severity of tics within the last week before the clinical evaluation (10). The total number of simple tics and total number of complex tics were counted for each patient based on a tic symptom checklist included in the YGTSS. To assess the lifetime intensity of tics we questioned about worst ever tic severity and qualified it as mild, moderate, or severe. Mild tics were defined as not related to physical or mental discomfort, problems in relations with peers, less than expected academic achievements and the need for treatment. Tics were assign as moderate if they generated only slight and temporary restrictions in the patients' daily lives (e.g., a few days' absence from school, as well as difficulties with homework). Tics classified as severe were those that caused an inability to continue normal daily activities (e.g., repeating grades, losing one's job or physical discomfort), a significant deterioration in the quality of life and the necessity of pharmacological therapy. We also collected information about the presence of premonitory urges.

To make a diagnosis of OCD, each patient was carefully questioned about obsessions and compulsions according to the checklist included in the Yale-Brown Obsessive Compulsive Scale (Y-BOCS). Then we analyzed if any of the present symptoms fulfilled the diagnostic criteria for OCD listed in the DSM-5. If a diagnosis of OCD had been made in psychiatric clinics before our evaluation, it was accepted and included into the analysis.

Different methods of data collecting were applied to children and adult patients. In contrast to children and adolescents, in whom most clinical information was provided by their parents, adults reported them themselves. All questions asked during the interview were part of routine practice and therefore no refusal rate is reported in this study.

### Study Participants

The cohort of GTS cases comprised of 195 consecutive patients aged 5–66 years (mean age: 15.0 ± 9.2; 47 females, 24.1%). The subjects were evaluated from 2013 to 2020. One hundred and forty children (71.8%, mean age: 10.3 ± 2.7) and 55 adults (mean age: 27.1 ± 8.8) were enrolled. The mean age of tic onset was 6.2 ± 2.7 years old. Duration of GTS in children ranged 1–13 years (mean 4.9 ± 2.6 years) and in adults 3–34 years (mean 17.0 ± 7.0 years).

### Statistical Methods

The statistical analyses were performed using Stata/SE release 14.2 (StataCorp LLC, College Station, Texas, USA). Data was presented as: (1) arithmetic means and standard deviation (mean ± SD) for continuous numerical variables; (2) medians and quartiles (Q_1_, Q_3_) for discrete numerical variables; (3) absolute numbers with frequencies (percentages) for categorical variables. Comparisons for two independent groups, considering the numerical traits, encompassed one-way analysis of variance without replication or the Mann-Whitney *U* test. Contingency tables, depicting and intersecting two categorical variables, were provided with the Chi-square test of independence with Yate's continuity correction when applicable. Regarding the statistical analyses carried out, their outcomes were considered significant when a two-tailed test returned *p* < 0.05. Gender and age were entered into the multivariate model as control variables.

## Results

At least one BT occurred at some point in the lifetime of 73 of 195 patients (37.4%), including 29 of 55 adults (52.7%) and 44 of 140 children (31.4%). BTs occurred significantly more often in adults than in children (*p* = 0.006). BTs were present in 48 patients (65.8%) at evaluation, and in 25 patients (34.2%) they were present only in the past. The age of onset was known in only 50 patients, and was 10.4 ± 5.9 years (range: 4–30 years). BTs occurred an average of 4.8 ± 5.3 years after tic onset. Most frequently, the onset of BTs was during childhood (76.0% at the age of 5–11 years), less frequently in adolescence (12.0% at the age of 12–17 years) and in adulthood (12.0% at the age ≥18 years). The most common BT was cessation of walking (*n* = 59, 80.8%), followed by speech (*n* = 19, 26.0%), running (*n* = 18, 24.7%), and writing (*n* = 9, 12.3%). Fifty-two patients (71.2%) reported cessation of one activity, 11 patients (15.1%) of two activities, three types of BTs occurred in nine patients (12.3%), and only one person (1.4%) had four types of BTs. The median of number of BT types was one (Interquartile Range IQR 1-2).

Univariate analysis showed a significant correlation between BTs and the worst ever tic severity, YGTSS, total number of simple tics, total number of complex tics, age at evaluation, and OCD (Table 1). Multivariate logistic regression analysis showed a significant association between BTs and the worst ever tic severity, and total number of complex tics (Table 2).

**Table 1:** Univariate comparisons between BTs+ and BTs- study groups (*n* = 195, missing data were deleted case-wise).

**Variable**	**Patients with BTs** ** (*n* = 73)**	**Patients without BTs** ** (*n* = 122)**	***p*-value**
Gender (females), *n* (%)	19 (26.0)	28 (23.0)	0.627
Age at evaluation (years), mean (SD), min.–max.	16.8 (9.4), 6–44	14.0 (8.9), 5–66	**0.042**
Worst ever tic severity, *n* (%)			
Mild	6 (8.2)	32 (26.2)	
Moderate	32 (43.8)	74 (60.7)	**<0.001**
Severe	35 (48.0)	16 (13.1)	
YGTSS, median (Q_1_-Q_3_), min.–max.	59 (47–72), 12–96	35 (20–54), 5–85	**<0.001**
Total number of simple tics ever experienced, median (Q_1_-Q_3_), min.–max.	12 (10–17), 5–25	11 (7–14), 2–24	**0.030**
Total number of complex tics ever experienced, median (Q_1_-Q_3_), min.–max.	9 (6–15), 1–30	5 (3–8), 0–26	**0.002**
Premonitory urges, *n* (%)	41 (59.4)	69 (63.9)	0.550
Obsessive-Compulsive Disorder, *n* (%)	24 (32.9)	20 (16.4)	**0.009**

*BTs, blocking tics; n, number; SD, standard deviation; min., minimum value; max., maximum value; YGTSS, Yale Global Tic Severity Score; Q_1_, lower quartile; Q_3_, upper quartile. Bold values are those statistically significant p < 0.05*.

**Table 2:** Multivariate logistic regression estimates (*n* = 195).

**Variable**	**OR**	**95% CI**	***p*-value**
Gender (females)	0.58	0.16–2.15	0.417
Age at evaluation (years)	1.03	0.97–1.09	0.378
Worst ever tic severity	**3.04**	**1.25**–**7.33**	**0.014**
YGTSS	1.01	0.98–1.05	0.492
Total number of simple tics ever experienced	0.96	0.83–1.11	0.596
Total number of complex tics ever experienced	**1.11**	**1.01**–**1.23**	**0.044**
Obsessive-Compulsive Disorder	2.30	0.45–11.69	0.455

*OR, odds ratio; CI, confidence interval; YGTSS, Yale Global Tic Severity Score. Bold values are those statistically significant p < 0.05*.

## Discussion

Among the patients with BTs, 2/3 had incidents present at evaluation and in 1/3 they occurred only in the past. Thus, BTs have a “waxing and waning” pattern, which is characteristic for classic tics. It can be inferred that BTs belong to complex tics based on the calculated correlation between BTs and the total number of complex tics, suggesting that several muscles must be involved to produce a BT. Additionally, BTs appeared on average a few years after tic onset which corresponds to observations that complex tics generally appear later than simple ones (11). We did not find a significant difference in the incidence of premonitory urges in the group of patients with BTs as compared to patients without BTs. Thus, the statistical analysis did not confirm our clinical speculation that BTs are slightly more automatic than other types of tics.

We analyzed primary and secondary BTs together, because it was not possible to differentiate them based on the interview. On the one hand, inhibition of performed activity may have two causes, even occurring together in the same patient. For example, cessation of writing may once be a primary BT and another time result from a sudden motor tic of the upper limb. On the other hand, different patients may report the same symptom which has a different cause in each of them. For example, gait arrest may be a primary BT in one patient, and in the other, cessation of walking may be secondary to the need to jump or squat. Our patients very rarely reported the occurrence of BTs spontaneously, in most cases we had to ask about their presence. This is probably because many BTs are secondary to other tics and for this reason patients do not distinguish them. It seems that if we were to only include primary BTs into the analysis, their prevalence would be much lower. Including patients with BTs secondary to other types of tics preceded by urges into the BT group may be a possible reason for not finding a significant difference in the incidence of premonitory urges in patients with BTs compared to patients without these tics.

Although we excluded patients with clear OCD-related motor blocks, we showed a positive correlation between BTs and the presence of OCD, but only in univariate statistical analysis (Tables 1, 2). Based on our findings, we believe that tic-related variables, compared to OCD, are more important correlates of BTs (Table 2). OCD is one of the most common comorbidities in GTS patients and the clinical picture of compulsions and tics overlaps. Therefore, it is possible that either we enrolled some patients with OCD-related motor blocks into our study group or OCD represents a putative risk factor for the development of BTs. Kwak and Jankovic described a patient in whom verbal blocking had a dual background: speech was delayed by completing a specific thought pattern in response to an obsession but also by completing a stereotypic motor tic (8). We can speculate that tic-related and OCD-related motor blocks may belong to the same spectrum of negative motor phenomena.

Patients with BTs had more severe tics, as assessed with the YGTSS, worst ever tic severity, and a greater overall number of tics (Tables 1, 2). This seems to prove that BTs are associated with a more severe form of GTS. Another argument supporting this theory is the coincidence of BT onset with the period of most severe tics. The severity of tics typically peaks at the age of 10–12 years and then tics subside significantly during adolescence (12). In our study, BT onset was just over 10 years of age. The overlapping of BT onset with the most severe period of the disease may suggest that this type of symptom can significantly add to the impairment caused by tics.

One of our hypotheses assumed that BTs were much more prevalent in GTS patients than previously thought and that a patient's age could be a variable influencing the incidence of BTs. This seems to be confirmed by the fact that patients with BTs were older at evaluation than patients without such tics, and that BTs occurred more often in adults than in children. They appeared in more than 1/3 of all GTS patients and, in about $\frac{3}{4}$ of patients, the onset of BTs was during childhood. Therefore, we can consider them as an early and common symptom of GTS.

We believe it is clinically important to be aware that the clinical picture of GTS include blocking phenomena. Otherwise patients with tics reporting sudden inhibitions of motor activity could be misdiagnosed as having functional disorders. It results from the fact that BTs do not fit the current definition of tics which basically applies to clonic tics. Similarly, tonic and dystonic tics also do not meet the classic criteria of tics and are therefore often underdiagnosed. As mentioned before, BTs are sometimes the result of prolonged tonic or dystonic tics. Thus, the presence of BTs may bring the clinician's attention to the presence of other atypical tics. It is important to diagnose accurately all patient's symptoms to correctly assess the total impairment caused by tics. This enables the clinicians to implement the tic-specific therapy when it is necessary.

Due to the fact that about half of GTS patients have comorbid OCD (13), it is important to differentiate between BTs and OCD-related blocking phenomena in GTS patients. In contrast to BTs which belong to involuntary movements, OCD-related blocking phenomena are performed voluntarily. In the latter case inhibitions of motor activity are caused by a sudden need to immediately perform certain activities in a planned, specific way. This results in the interruption of the current activity in order to perform the set of mental or motor compulsions. In OCD patients refraining from performing these ritualistic behaviors leads to increased tension and anxiety while BTs are not anxiety-related. Another difference between the two discussed phenomena is that BTs are usually short lasting while OCD-related cessations of motor output last longer as performing compulsions is often time-consuming. The above differential diagnosis is essential because both pharmacological treatment and behavioral interventions are different for tics and OCD.

It is worth noting that phenomenologically, phonic BTs may resemble stuttering. Van Borsel et al. analyzed speech patterns in patients with GTS and found that they do not meet the criteria of any known and well-defined speech disorders but are similar to stuttering, cluttering and palilalia (6, 7). In patients with GTS stuttering is considered fairly common, with incidence varying according to different studies, from 15.3 (14) to 31.3% (15). Perhaps in some cases these are misdiagnosed BTs. It is clinically important to differentiate genuine stuttering from tic-related cessations of speech because it influences the choice of the therapeutic methods employed. It seems that speech therapy, although recommended for stuttering, does not improve BTs in which only tic-specific therapy is effective (5).

We believe that our study proves that BTs, although not widely investigated so far, are an important part of GTS clinical picture and therefore require further research. It seems that clinicians should actively investigate whether inhibitions of motor activity occur in their GTS patients. Perhaps a useful tool would be to create questionnaires concerning atypical tics to collect information on their prevalence, duration, age of onset, and correlation with other tics. Using such a tool in everyday practice could allow to correctly diagnose more patients and would facilitate future research on tics that do not fit the classic definition.

The most important limitation of the performed study was recall bias. Some patients did not remember whether they had ever experienced any cessations of motor activity, the exact phenomenology of symptoms or the age of onset. This applies in particular to adult patients in which BTs occurred in childhood and to patients with secondary BTs as such patients were often unaware of their inhibitions of motor activity. Only in a few cases family members provided additional useful information. Another limitation of the study was that 1/3 of patients with BTs reported presence of BTs only in the past. In these cases, it was not possible to objectify the reported symptoms because patients were not assessed by a specialist at the time of BTs appearance and only in few cases video recordings of reported symptoms were available. This might have influenced the rate of BTs in our GTS cohort and therefore our findings should be re-evaluated in future studies.

## Data Availability Statement

The raw data supporting the conclusions of this article will be made available by the authors, without undue reservation.

## Ethics Statement

The studies involving human participants were reviewed and approved by the Ethics Committee of the Medical University of Warsaw (KB/2/2007). Written informed consent to participate in this study was provided by the participants' legal guardian/next of kin.

## Author Contributions

JK: design, execution of statistical analysis, and writing of the first draft of manuscript. PJ: conception, organization, and execution of research project, design and review and critique of statistical analysis, and review and critique of manuscript. Both authors contributed to the article and approved the submitted version.

## Conflict of Interest

The authors declare that the research was conducted in the absence of any commercial or financial relationships that could be construed as a potential conflict of interest.
